# Call for Papers for MSM 2012 Theme Monograph

**Published:** 2011

**Authors:** Ajai Singh

**Affiliations:** *5 Jan 2011 Editor, Mens Sana Monographs, Medicine. Mental Health. Man, Mind And their Matrix, mensanamonographs@yahoo.co.uk*

## Mind, Consciousness and the Brain: Contributions from Indian Thought

Following closely upon the heels of the MSM 2011 Theme Monograph titled, *’Brain, Mind and Consciousness: An Interdisciplinary International Perspective,* is this Call For Papers for another Theme Monograph in 2012 which seeks to present the views of Classical and contemporary Indian thinkers on the topics of Mind, Consciousness and Brain. Interested scholars and researchers may choose topics from the list below:

## Topics*

1. Concept of Mind and Consciousness in the Indian Philosophies: An Overview
2. Relevance of Indian Concept of Mind and Consciousness to World Philosophy
3. Analytical study of the concept of Mind in the Indian Philosophies
4. Comparative study of Mind in Indian and Western thought
5. Mind in the different *darśanas*
6. Mind in the *Upanisads*
7. Is Indian Thought on Mind and Consciousness Relevant Today?
8. Jaina concept of Mind and Consciousness
9. Mind and Consciousness in *Carvāka* thought
10. Nyāya concept of Mind and Consciousness
11. Mind and Consciousness according to Sri Aurobindo
12. Mind and Consciousness for Rabindranath Tagore
13. Phenomenal reality (*prāñibhāsika-sattā*), empirical reality (*vyāvahārika-sattā*), and absolute reality (*pāramārthika-sattā*)
14. *Vedānta,* Mind and Consciousness
15. Transcendental consciousness as “one only without a second” (*ekameva advitīyam*).
16. Advaitic concept of mind and consciousness
17. Buddhist concept of mind and consciousness
18. Samkhya concept of mind and consciousness
19. Mind and Consciousness for Swami Vivekananda
20. Mind, Consciousness and Sri Krishnamurti
21. Gandhi on Man, God and Consciousness
22. Modern Indian Thinkers on Mind and Consciousness
23. K.C. Bhattacharya and S. Radhakrishnan on Mind and Consciousness
24. Mind and Consciousness in Indian Thought of the last two decades 1990-2010.
25. Mind for Acharya Rajneesh
26. The Future of Indian Thought on mind and consciousness
27. Mind and Consciousness in the *Brahma-sūtra* of *Bādarāyana*
28. The state of *Sthitapragña*
29. Mind and Self in Indian thought
30. *Prājña* of the deep-sleep state, *Taijasa* of the dream state, *Viśva* of the waking state
31. Self above matter
32. *Tajjalān* and *kalpita*
33. *Brahman* and *Ātman*
34. Ego (*aham*) and *cidābhāsa,* i.e. consciousness reflected in the internal organ
35. Mind not identifiable with Self according to Indian thought
36. *Gaudapāda’s* declaration, *“upadeśād-ayam vādah* and *“jñāte dvaitam na vidyate”*
37. Brahman/*Ātman* neither immanent nor transcendent
38. Brahman/*Ātman* both immanent and transcendent
39. Empirical-relational objects with class feature (*jāti*), quality (*guna*), action (*kriyā*), or relation (*sambandha*), and signified by a conventional word (*rūdhi*)
40. The knower (*pramātā*), and the Self
41. Negative scriptural concepts like *“neti neti”*
42. Secular and sacred *śabda*
43. Ultimate reality trans-empirical and trans-relational
44. *Antahkarana* as internal sense organ
45. The concept of *manas*
46. *Jiva, manas* and *ātman*
47. *Vasanā, vairāgya* and *manas*
48. The state of *sat-cit-ānanda*
49. Knower (*jñātā*), “I” (*aham*) and “this” (*idam*).
50. Witness-consciousness (*sāksi-caitanya*),
51. *Pramāna* and *apramāna*
52. Distinguishing valid cognition (*pramā*) from erroneous cognition (*ābhāsajñ āna*)
53. Consciousness, as self-established (*svatassiddha*) and self-luminous and the transcendental *a priori*
54. *Upanisadic* theory of three worlds
55. Human being as material (*jada*) excepting the Self or Consciousness
56. Mind a sentient entity carrying the reflection (*pratibimba*) or semblance (*ābhāsa*) of Consciousness
57. The five organs of perception, the five organs of action [*karmendriyas*], the five vital breaths [*pranas*]
58. The mind [*manas*], intellect [*buddhi*], egoity [*ahamkāra*] and the mind-stuff [*citta*]
59. Waking experience (*jāgrat*), the world of dream experience (*svapna*), and the world of deep sleep experience (*susupti*)
60. *Upanisadic* tradition and the Fourth (*caturtha*) beyond the three worlds in 59 above.
61. Consciousness (*cit*) and experience (*anubhava*)
62. *Viśva, Taijasa* and *Prājña*
63. Triple Stream of Experience (*avasthā-traya*)
64. “I” as knower (*jñātā*), as doer (*kartā*), as experiencer (*bhoktā*)
65. *Jiva* and its *kośas*
66. The *Kośas: Annamaya* [sheath of food and matter], *pranamaya* [sheath of vital breath], *manomaya* [mental sheath], *vijñānamaya* [intellectual sheath] and *ānandamaya* [the sheath of bliss], and what do they signify in understanding the Self
67. Mind empowered with cognition of other objects, sense of “I” and “mine ”, and also self-conscious when need arises
68. Self-conscious mind and *jīva*
69. Self or foundational Consciousness
70. Self and the Mind
71. *Śaïkara* and *jñāna-karma-adhikāra*
72. Consciousness as support (*adhisthāna*) of objects of the entire world
73. Advaita *Vedānta* characterised as *“*transcendental phenomenology*”* and *“*metaphysics of experience*”*
74. Advaita as both pluralistic and monistic
75. *Citta* and *samskāras*
76. *Buddhi, ahamkāra* and *citta*
77. *Patanjali Yoga* and the eight fold path
78. Buddha’s four noble truths and eight fold path
79. *Citta-vrtti-nirodha*: how does it relate to the concept of Mind in Indian thought
80. Citta and vritts
81. *Ahamkāra* [or egoism] and the Mind
82. The state of mindlessness
83. The state of *moksa*
84. Kaivalya, Nirvana, Apavarga, Nihśreyasa
85. The concept of liberation in the Indian philosophies
86. *Ātman* and the Mind
87. Configuration (*avasthā*), place (*deśa*), time (*kāla*), and qualities (*guna*)
88. The concept of brain in Indian thought
89. Ayurveda, mind and brain
90. Body represented by the brain, mind represented by *vijñāna* and *ātman* represented by the life principle as making for the complete man
91. The state of *savikalpaka* and *nirvikalpaka samādhi*
92. The *Gunas - Sattva, rajas, tamas* - and the self
93. Advaita as affirming monism without denying pluralism
94. Naiskarmya-siddhi of Sureśvara.
95. *Buddhi* or cognition
96. The concept of *Citta*
97. The concept of *drsti*
98. The *Indriyas, Karmendriyas,* and *Jñānendriyas*
99. *Jñāna* or knowledge
100. *Smrti* or memory
101. Absolute Consciousness or *turīya*
102. Mind as an internal organ of sense
103. Mind as self
104. Mind as not the self
105. Mind as minute and subtle
106. Mind as instrument of knowledge
107. Mind as instrument of the soul
108. Self-cognition of Mind
109. Mind as cause
110. Mind and dream experience
111. Mind as reduced to a machine
112. Sense organs and mind contact
113. *Vrtti* or mental mode
114. Self or *Ātman* or Soul
115. Self as pure consciousness
116. *Vijñāna* or discrimination
117. *Prajñā* or intelligence
118. *Sannikarsa,* or relation between mind, sense-organ and the object
119. *Samkalpa* or power of conception

[*Kindly see to it that Sanskrit words are italicized and with proper diacritical marks in your paper.]

Authors must convey their topics selected from the above by 15^th^ April 2011. They maybe more than one topic for one paper, but not more than three. Please check topic availability with the Editor. For topics different from the above, contact Editor. Full paper for potential publication should reach the Editor in Microsoft Word format by 15^th^ July 2011.

- All papers will be submitted for peer review and a decision of acceptance or otherwise will be conveyed to the authors by 15^th^October 2011, or one month of receipt, whichever is later.
- Authors may contact the Editor, Mens Sana Monographs, for further details and clarification. Email:(mensanamonographs@yahoo.co.uk).
- Please check style requirements from recent issue of MSM, or at http://www.msmonographs.org/contributors.asp

